# Future Prospects for Scaffolding Methods and Biomaterials in Skin Tissue Engineering: A Review

**DOI:** 10.3390/ijms17121974

**Published:** 2016-11-25

**Authors:** Atul A. Chaudhari, Komal Vig, Dieudonné Radé Baganizi, Rajnish Sahu, Saurabh Dixit, Vida Dennis, Shree Ram Singh, Shreekumar R. Pillai

**Affiliations:** Center for Nanobiotechnology Research, Alabama State University, Montgomery, AL 36104, USA; atulvet@gmail.com (A.A.C.); komalvig@alasu.edu (K.V.); drbaganizi@gmail.com (D.R.B.); sahu.rajnish@gmail.com (R.S.); sdixit@alasu.edu (S.D.); vdennis@alasu.edu (V.D.); ssingh@alasu.edu (S.R.S.)

**Keywords:** scaffolds, biomaterials, synthetic, natural, polymer, skin, wound healing, tissue engineering

## Abstract

Over centuries, the field of regenerative skin tissue engineering has had several advancements to facilitate faster wound healing and thereby restoration of skin. Skin tissue regeneration is mainly based on the use of suitable scaffold matrices. There are several scaffold types, such as porous, fibrous, microsphere, hydrogel, composite and acellular, etc., with discrete advantages and disadvantages. These scaffolds are either made up of highly biocompatible natural biomaterials, such as collagen, chitosan, etc., or synthetic materials, such as polycaprolactone (PCL), and poly-ethylene-glycol (PEG), etc. Composite scaffolds, which are a combination of natural or synthetic biomaterials, are highly biocompatible with improved tensile strength for effective skin tissue regeneration. Appropriate knowledge of the properties, advantages and disadvantages of various biomaterials and scaffolds will accelerate the production of suitable scaffolds for skin tissue regeneration applications. At the same time, emphasis on some of the leading challenges in the field of skin tissue engineering, such as cell interaction with scaffolds, faster cellular proliferation/differentiation, and vascularization of engineered tissues, is inevitable. In this review, we discuss various types of scaffolding approaches and biomaterials used in the field of skin tissue engineering and more importantly their future prospects in skin tissue regeneration efforts.

## 1. Introduction

Wounds are defined as disruption of any tissue or cellular integrity due to mechanical, physical or metabolism (mainly due to diabetes mellitus) related injuries. In response to the injury or as a recovery or healing process, the major priority is to stop hemorrhage, to avoid excessive blood loss, and prevent microbial infection by infiltration of immune cells, such as neutrophils or macrophages. More importantly, it is critical to restore the function of the damaged tissue or cell through rapid healing. Wound healing is a stepwise process which includes (1) an inflammatory stage characterized by macrophage or leucocytes infiltration and cytokine production; (2) a proliferative phase which includes removal of damaged tissue and formation of granulation tissue in the wound; (3) a maturation phase wherein extracellular matrix produced by the proliferative tissue becomes well-defined; and (4) the formation of scar tissue indicating the completion of the wound healing process [1,2]. The process of wound healing is more or less similar in all types of tissues, including skin tissue. In particular, skin tissue wounds are categorized as epidermal, dermal or dermo-epidermal, based on the degree and intensity of such wounds [2]. The molecular mechanism of skin wound healing mainly involves production of various growth factors, such as epidermal growth factors (EGF) and tissue growth factors alpha and beta (TGF-α, TGF-β), etc. The conventional approaches used for instant healing of skin wounds include the use of natural products that have anti-inflammatory, anti-microbial and antioxidant properties, such as turmeric (active component curcumin), honey, etc. Hot or cold fomentation at the wound site may reduce inflammation and fasten the healing process [1,3]. In the recent few years, the field of regenerative tissue engineering has emerged as a gold standard platform for the development of artificial tissues and organ regeneration, to resolve major health related issues in humans [4]. Multiple disciplines, such as cell biology, biomaterial research, bioengineering, etc., have contributed to the flourishing advances of tissue engineering. The major principle of tissue engineering is to restore and improve the function of the tissues by either generating novel or biocompatible substitutes or by reconstruction of the tissues [4,5]. Use of cells or cell implants, delivery of tissue growth enhancing factors and use of various matrices, such as scaffolds to generate three dimensional (3D) cellular structures are the three major pioneering approaches of tissue regenerative medicine [4,5,6]. In this review, we discuss one of the above triads of tissue engineering, i.e., scaffold types, and materials used for the fabrication of scaffolds, their advantages and disadvantages, with a major focus on applications in skin tissue engineering. Several scaffolding approaches have been reported for their efficient use as suitable matrices to facilitate the growth and differentiation of skin cells [2,7,8,9]. In this paper, we review various scaffolding approaches, types of scaffolds and various biomaterials used in skin tissue engineering and wound regenerative medicine.

## 2. Regenerative Skin Tissue Engineering and Wound Healing Using Various Skin Substitutes

Skin serves as an important organ in the human body’s “first line of defense” system and is primarily involved in protection against the outside environment and thermoregulation [9]. It is composed of an outer epidermis and inner dermis layer; each layer with specific functions, such as prevention of dehydration, a barrier to avoid trauma, sensory perception, vitamin D synthesis and immune surveillance. The outer layer of the epidermis mainly consists of keratinocytes (almost 90%) which eventually proliferate from the basal layer and differentiate at the terminal layer of the epidermis to form the cornfield epidermal layer. The epidermis is a highly regenerative layer of the skin due to the presence of stem cells which contribute to the homeostasis of the skin and eventually help in the process of wound repair. Wound healing and thereby regeneration of the skin tissue is a very well-coordinated process and depends upon several factors, such as wound type (epidermal, deep dermal, full thickness), tissue damage by either burn (first, second and third degree) or physical trauma, moisture conditions surrounding the wound, inflammation, secondary infections, etc. [1]. The various steps involved in skin wound healing are formation of new cells through secretion of the extracellular matrix (ECM) by fibroblast cells followed by keratinocyte formation and proliferation in layers and finally the differentiation of keratinocytes to form the outermost layers of the epidermis, such as stratum lucidum and stratum corneum. Most of the minor wounds are healed by simple contraction and growth of the cells inside the wound. However, the large skin wounds take much longer to heal and hence are more prone to risks, such as inflammation, infection, scar formation, etc., which results in the formation of chronic wounds [1]. Some of the factors, for example, disease conditions such as diabetes, renal infections, presence of foreign bodies, malnutrition and immunocompromised status of the body and older age affect the process of normal wound healing and thereby tissue restoration. Therefore, it is extremely important to include these factors while engineering various grafts in the field of skin tissue regeneration [1,4,7,9]. Over the past several years, skin tissue regeneration has shown promise due to the invention of several novel skin tissue engineered products. Various (allo, auto, xeno, etc.) grafts of dermal, epidermal or dermo-epidermal origin have been reported and have been used commercially [2,8,9]. Such grafts help restore the structure of the skin tissue by repairing the wound effectively. Such bioengineered skin substitutes not only repair the wounds, but also have various supplements, such as growth factors, antibiotics and anti-inflammatory drugs which eventually fasten the wound healing process. To engineer these substitutes, various scaffold matrices have been developed to promote cell growth in 3D structure. Such scaffolds are highly biocompatible with skin tissue and biodegradable in nature, acting as suitable dressing material for wound healing. Recent advances in the skin tissue engineering field revolve around the use of scaffolds with cell population, such as keratinocytes and fibroblasts [8].

## 3. Scaffolding Approaches and Different Types of Scaffolds in Skin Tissue Engineering

**Figure 1:**
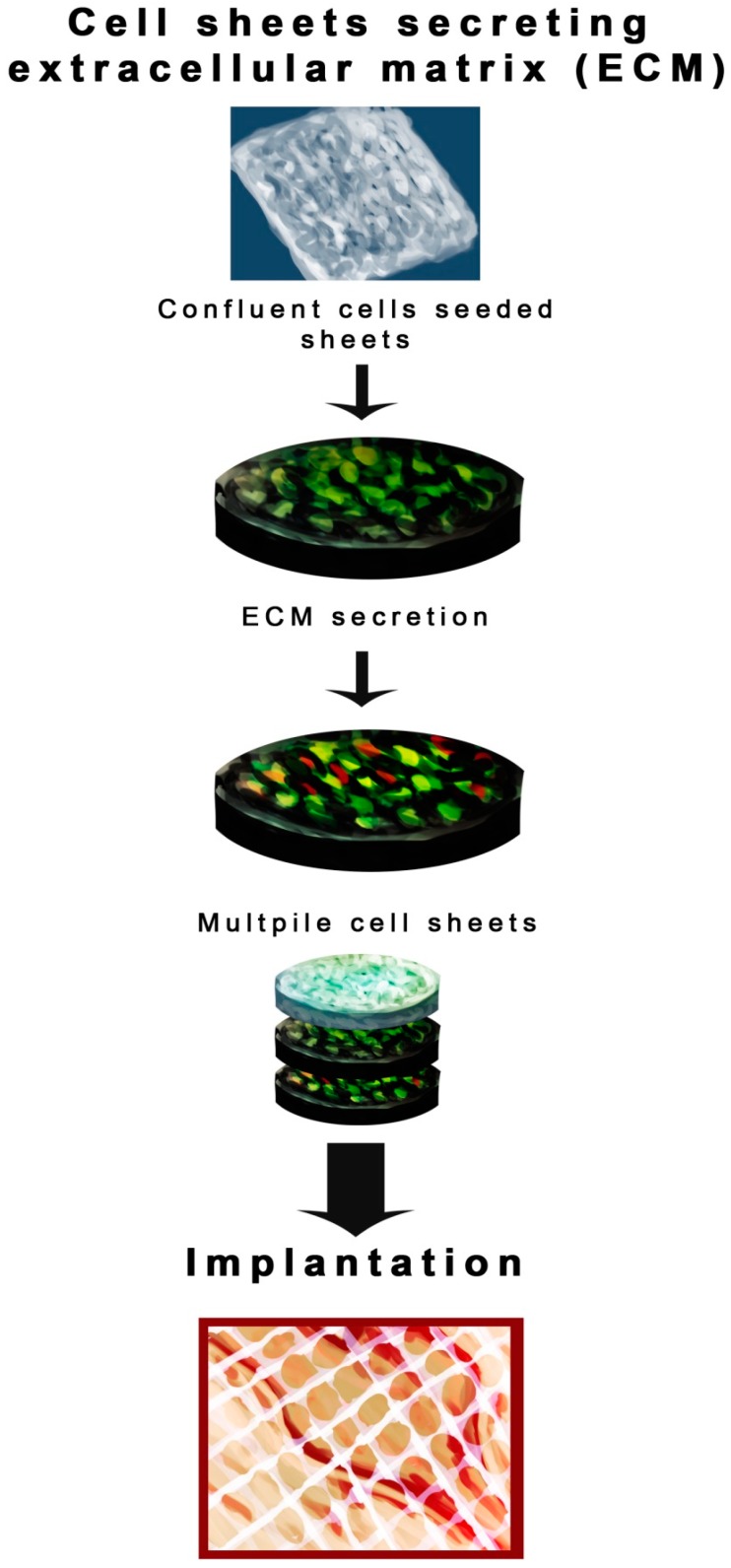
Cell sheets that secret extracellular matrix (ECM). Cells are seeded on the sheets and allowed to secret ECM that facilitates growth and proliferation. Multiple cell seeded sheets capable of secreting ECM are used for implantation at the wound site.

**Figure 2:**
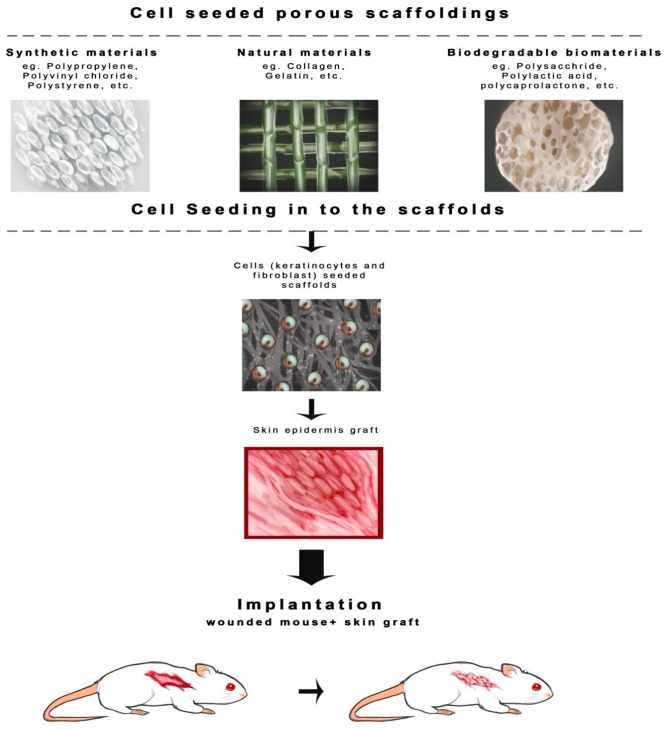
Porous scaffolding using various biomaterials. Various natural, synthetic and biodegradable materials are used for generation of highly porous scaffolds. These scaffolds provide a suitable environment for cell growth and proliferation. The porous nature of such scaffolds facilitates the regular supply of nutrients and oxygen for the skin cells, such as keratinocytes and fibroblasts. The full thickness skin grown on such scaffolds is used for wound transplant.

**Figure 3:**
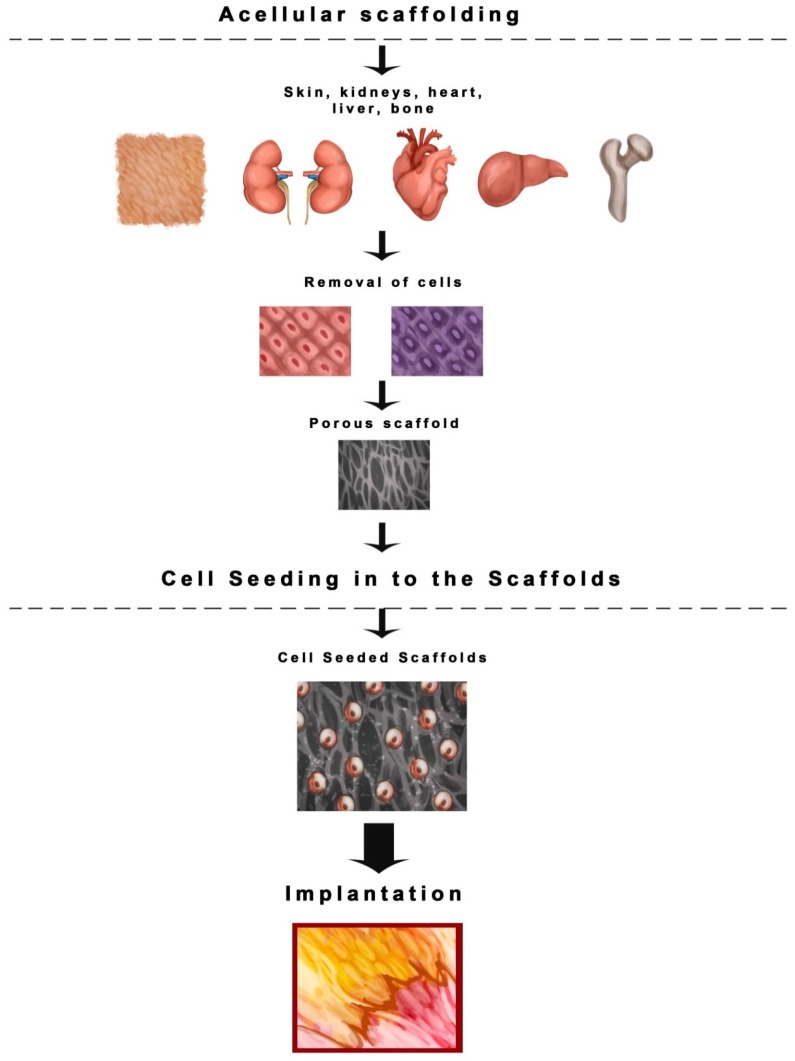
Acellular scaffolding approach. In this approach, complete de-cellularization of the organ is performed to create extracellular (ECM) based matrices. The cells of interest, such as skin cells, liver cells or any other organ specific cells can be then effectively grown on such scaffolds.

**Figure 4:**
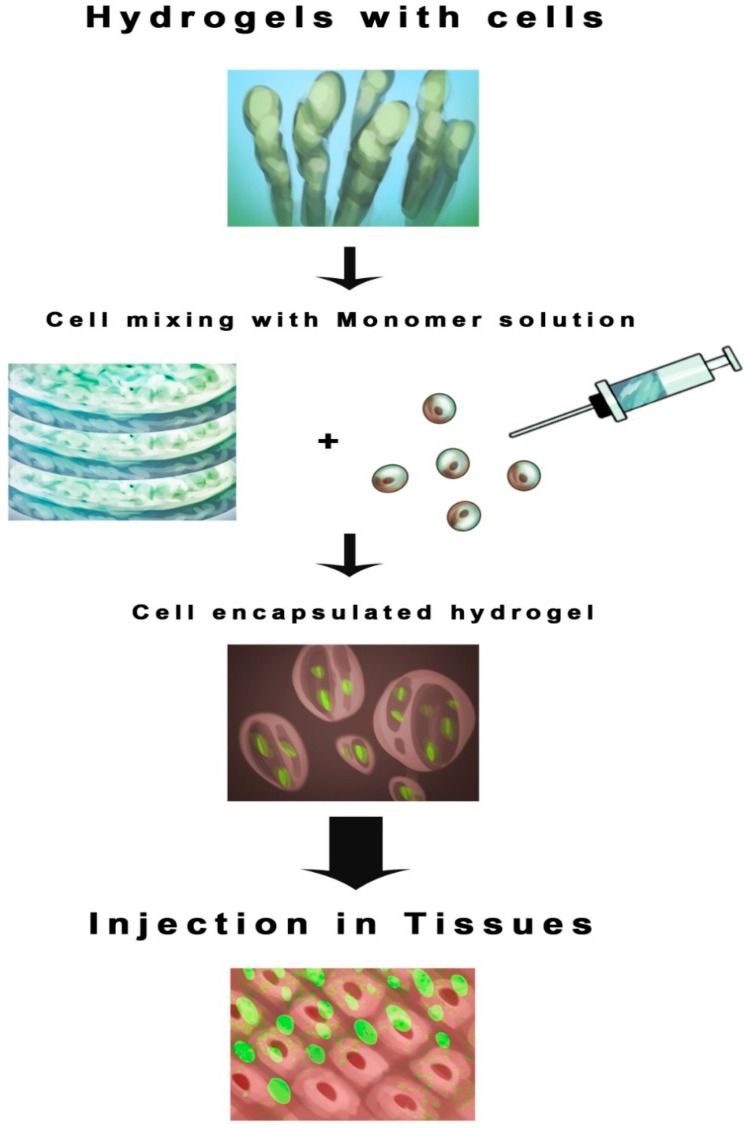
Hydrogel approach. Monomer mixture of a polymeric solution, for example, polyethylene glycol, poly-caprolactone, chitosan, cellulose, etc., are mixed with the skin cells, such as keratinocytes and fibroblasts to generate injectable hydrogels at the wound sites to facilitate wound healing and skin regeneration.

Among the recent technologies in the multidisciplinary field of tissue engineering or regenerative medicine, use of various types of scaffolds is the key component. As per the sophisticated definition of tissue engineering described at a National Science foundation workshop, scaffolds are the best materials for restoring, maintaining and improving tissue function [10]. They play a unique role in repair and more importantly regeneration of tissues by providing a suitable platform, permitting essential supply of various factors associated with survival, proliferation and differentiation of cells [5,11]. Scaffolds can be made up of synthetic or absorbable, naturally occurring, biological, degradable or non-degradable polymeric materials [12,13,14,15,16,17,18,19,20]. Several techniques have been used to construct scaffolds [21,22,23,24,25,26,27,28,29,30,31,32,33,34,35,36,37] but the four major scaffolding approaches [6] include the use of ECM secreting cell sheets (Figure 1) [38,39,40], pre-made porous scaffolds of synthetic, natural and biodegradable biomaterials (Figure 2) [41,42,43,44,45,46], decellularized ECM scaffolds (Figure 3) [46,47,48,49], and cells entrapped in hydrogels (Figure 4) [50,51,52,53]. All these approaches have advantages as well as drawbacks. In this review, we intend to focus on the different types of scaffolds based on their biomaterial design and their advantages/disadvantages, especially those scaffolds which are extensively used for skin tissue regeneration (Table 1). As shown in Figure 1, the cells are seeded on the thermally regulated polymeric materials which are made up of poly(*N*-isopropylacrylamide), material with a lower critical solution temperature (LCST) of 25 °C, and then allowed to grow to full confluency to produce cell sheets that secrete ECM. These cell sheets then can be easily separated from the polymer surface without trypsinization, by simply lowering the incubation temperature below 25 °C [6]. Such cell sheets secreting ECM can then be used for transplantation (Figure 1). Based on the four approaches mentioned above, the different types of scaffolds currently in use are as follows:

### 3.1. Porous Scaffolds

Porous scaffolds exist in different forms, such as sponge, foam, mesh, and nano- and microscale biodegradable fibers; the last two types can indeed be categorized under fibrous scaffolds (Figure 2) [54,55]. Fabrication of porous scaffolds is generally performed by (1) use of porogens to monitor the desired size and shape of the pores in biomaterials; (2) prototyping; (3) layer-by-layer cell and woven or non-woven nanofibers by electrospinning technology; and (4) the most recent 3D printing [6,8]. Figure 2 depicts the fabrication of the porous scaffolds using either biodegradable synthetic, natural or composite biomaterials. The porous nature of the scaffolds allows seeding of the cells and proper exchange of nutrients and such cell seeded porous grafts are then used for implantation into the hosts (Figure 2). They have been used for growth of tissues, organs and hard tissues such as bone [56]. These scaffolds have interconnected pore networks with greater porosities to simulate extracellular matrix construction for effective interaction of the cells with their environment. Foams and sponges are mechanically more stable compared to mesh structures. Porous scaffolds provide a physical surface for cells to bind and produce their own ECM and more importantly provide improved nutrient supply to the center of the device through interconnecting pores, thus reducing the chances of developing a necrotic center [5]. An ideal porous scaffold has a specific pore size with greater porosity and an appropriate surface-to-volume ratio that enables diffusion of the nutrients, drug, etc. [57,58]. Some of the synthetic biodegradable polymers used as porous scaffolding materials include poly-ethylene glycol (PEG), poly(lactic acid) (PLA), polyglycolide (PGA), poly(lactic-*co*-glycolic acid) (PLGA) [56], polycaprolactone (PCL) [59], poly(d,l-lactic acid or d,l-lactide) (PDLLA), polyester elastomer (PEE) based on polyethylene oxide (PEO), and Polybutylene terephthalate (PBT) [28]. We will be discussing various natural as well as synthetic biomaterials that are used for scaffold design in the later part of this review. 

An ideal porous scaffold in skin tissue engineering is the one that mimics the natural environment for skin growth through appropriate cell infiltration, proliferation and differentiation. It should be biodegradable, permeable to oxygen, water and nutrient exchange and should be protective against infection and mechanical damage [55,60,61,62,63,64]. To date, there have been several forms of porous scaffolds described for skin tissue regeneration [54,55] and most of them can be categorized as fibrous porous scaffolds. However, there are several spongy or foamy scaffold types with higher porosity which can be used as effective bio-constructs for skin regeneration [2]. Most of these porous scaffolds have collagen as a base and then keratinocytes or fibroblasts are seeded into the scaffolds. Orcel^®^ (Ortec International, New York, NY, USA) is a tissue engineered skin construct made up of bilayered type-I bovine collagen sponge which is porous at the base [2,65]. The bilayer structure of the sponge has a top layer of non-porous collagen-gel, on top of which keratinocytes are added and the layer beneath is porous collagen sponge where fibroblasts are seeded. Orcel^®^ has been approved by the food and drug administration (FDA) to treat donor sites in burns. The collagen sponge is capable of producing several growth factors that facilitate wound healing. In general, it is well tolerated, and promotes faster healing with minimum irritation compared with the acellular wound dressing Biobrane^®^ [65]. Yet another collagen sponge-based promising skin construct has keratinocytes and fibroblasts cultured on a collagen sponge to create cultured skin substitutes for clinical use, however this product is currently not available commercially [66,67]. The two sponge scaffolds discussed above are mainly dermo-epidermal composite skin substitutes [2]. There is an exclusively dermal substitute made up of bovine collagen sponge named as Terudermis^®^ in which the collagen sponge is bound to a silicone layer similar to Integra^®^, another dermal substitute of the fibrous scaffold type. This material is designed for the treatment of deeper burns [68] and is useful in regeneration [69] and to correct trauma induced deformities [70]. Besides these porous sponge scaffolds which are made up of bovine collagen, a sponge scaffold made up of collagen of porcine origin is the main constituent of Pelnac^®^ Standard Type scaffolds by Gunze Ltd., Kyoto, Japan. These scaffolds are indicated in acute burns and skin defects induced due to the removal of tumors or skin grafting in patients undergoing necrotizing fasciitis due to bacterial infections [71,72,73]. In addition, some novel sponge scaffolds in combination with biomaterials, such as human keratin and polyvinyl alcohol/chitosan have also been reported for their use as effective skin substitutes [74,75].

### 3.2. Fibrous Scaffolds

As mentioned above, fibrous scaffolds can actually be categorized as porous scaffolds and are made up of nanofibers and have a great potential to mimic the natural environment for human tissue (Figure 2). Nanofibers are synthesized using techniques such as self-assembly, phase separation, drawing, template synthesis and the more widely used electrospinning technique [56,76,77]. The nanofibrous structures combined with their porosity provide a highly desired niche for seeded cells for adhesion, proliferation and differentiation [30,78]. Nanofibrous scaffolds are widely used for hard as well as soft tissue engineering applications and they also act as vehicles for the controlled delivery of drugs and various biological molecules in the form of proteins and DNA [79]. Several natural and synthetic polymers have been utilized for nanofiber fabrications to produce fibrous scaffolds for biomedical applications [80,81,82,83,84,85,86,87,88,89,90]. These nanofibers are sometimes specifically functionalized by a simple blending (or mixing) or coating technique, or by surface grafting polymerization for attaching ligand molecules and adhesive proteins on the nanofiber surface. Blending of drugs, growth factors, and genes directly into the polymer solution during electrospinning is also practiced for controlled release properties [91]. More recent approaches for incorporating therapeutic agents or bioactive molecules include co-axial electrospinning, emulsion electrospinning, and modifications via surface absorption or chemical conjugation. [92,93].

As far as the skin tissue engineering field is concerned, a recent review on electrospun scaffolds discusses, in depth, the recent advances in skin generation through the use of electrospun nanofiber-based scaffolds [8]. Amongst the several scaffolds described in the dermal tissue regeneration, highly porous nanofibrous scaffolds have received much attention due to their (1) fibrillar structural resemblance to ECM; (2) efficiency for facilitation of nutrients exchange and removal of metabolic waste; and (3) ability to restrain the penetration of microorganisms due to small pore size [94,95]. There are plenty of natural polymers being used for nanofibrous scaffold fabrication with applications as skin substitutes and wound dressings [2,8]. Chitosan nanofibrous scaffolds have been observed to work better than 3D sponges of chitosan in terms of adhesion, growth and differentiation of keratinocytes, fibroblasts and endothelial cells. Recently, the use of bioceramic ultrathin fibers has been reported for their use in skin repair [96]. Some commercially available fibrous scaffolds that have been reported include Terudermis^®^ and Pelnac^®^ (in combination with collagen sponge), Biobrane^®^ and Integra^®^ as acellular scaffolds, and Apligraf and Transcyte as cellular scaffolds [2,3,8]. 

### 3.3. Acellular Scaffolds

Collagen rich matrices created by the removal of cellular components of the tissues are popular for their use to manufacture acellular scaffolds (Figure 3) [97,98,99]. When implanted on wounds, such scaffolds degrade slowly and are mostly replaced by ECM proteins produced by the growing cells. The design of such scaffolds should ensure that the biological activity and integrity of the ECM should not be affected adversely upon removal of the cells. As shown in Figure 3, cells are removed from the tissues such as kidneys, skin, etc., and the decellularized material could be used as a porous scaffold to seed any kind of cells to grow, allowing such implants to be used for various purposes. Due to the closeness of the decellularized material to the natural components of tissues and organs, such scaffolds may be useful for successful transplantation (Figure 3). Acellular tissue scaffolds have shown promising regenerative effects in genitourinary tissues without immunogenic rejection [97,100,101]. Such scaffolds are fabricated by using decellularization techniques based on either chemical, physical or enzymatic degradation. Such techniques include repeated freeze-thaw cycles, hypertonic or hyptonic solution treatment, trpsin/EDTA treatment, etc. This allows the biochemical composition and texture of the decellularized material to be maintained as close to its natural form as possible, so that the material can be used as an effective substitute to grow the damaged tissue [6,8]. Compared to other scaffolds, acellular scaffolds are advantageous in terms of retaining the ECM architecture with intact cell adhesion ligands. This facilitates tissue growth that is similar to native tissue, and more importantly reduces the immunologic responses to the grafts, thus ensuring their long term functionality [5]. Acellular scaffolds based on various extracellular matrices have been approved for their use in human tissue regeneration, such as for regeneration of heart, intestines or urinary bladder [102].

In the skin regeneration field, several dermal and epidermal acellular scaffold-based grafts have been reported [7]. The de-epithelialized acellular dermal allografts are prepared by removing cells and infectious and antigenic components [7,103]. The acellular dermal substitutes are produced by using natural and synthetic polymers or a combination of both. Natural polymers are usually the constituents of human dermal ECM, such as collagen, elastin, fibronectin, chitosan, etc. [104,105,106,107,108,109,110,111,112,113,114,115,116,117,118]. Although they are less toxic and produce low inflammatory responses, the biostability of natural polymers is very poor, therefore, limiting their strength and facilitating wound contraction [114,119]. This can be rectified by cross-linking of natural polymers to other natural or synthetic polymers [114,119]. However, for wound healing purposes, such cross-linked polymers are not useful because cross-linked polymer scaffolds offer great durability but limited cell infiltration, which is the most important factor for wound healing [120,121]. Synthetic polymers used for acellular scaffold fabrication can be categorized as absorbable synthetic polymers, such as PCL, PLA, PEG, etc., and nonabsorbable synthetic polymers including polyurethane, nylon, polytetrafluoroethylene (PTFE), etc. [114,118,122]. Synthetic polymers are relatively cheaper and offer the desired mechanical strength but they have tissue compatibility issues [118]. The different types of acellular scaffolds used in skin tissue engineering are discussed in a recent review article [7]. Several commercially available acellular scaffolds have been described as effective skin substitutes for wound healing [7,9]. Acellular allografts include Alloderm^®^, DermaCELL^®^, DermaMatrix^®^, FlexHD^®^, Graftjacket^®^, Graftjacket Xpress^®^ and Integra^®^ and Aplicaf^®^ (both bovine), whereas xenografts such as EZderm Mediskin^®^, OASIS Ultra^®^, MatriStem^®^ and MicroMatrix^®^ (all porcine) are also available for wound healing applications [7,9]. However, the xenografts are usually cross-linked with chemicals which limit their application in wound healing. Permacol^®^ is one such xenograft which has since been abandoned, whereas some other xenografts have not shown any promising results [7,123,124,125,126,127,128,129,130,131,132,133,134]. 

### 3.4. Scaffolds Based on Hydrogels

Hydrogel scaffolds which are made up of naturally derived macromolecules [135,136,137,138,139,140] or synthetic polymers [141,142] have a great potential due to their biocompatible and biodegradable nature and their ability of intrinsic cellular interaction (Figure 4). Recent developments in the design and application of biodegradable hydrogels led to dramatic advancement in controlled drug delivery and tissue engineering [143]. To guide the growth of new tissues, hydrogels comprised of synthetic polymers have potential advantages over hydrogels made up of natural macromolecules. As Figure 4 indicates, the monomeric solution of synthetic polymers can be mixed with the cells and the cell encapsulated hydrogels are available for injectable use in the tissues. Such hydrogels are prepared by ionic or covalent crosslinking of different polymers which are biocompatible to facilitate the encapsulation of living cells or drug molecules. One of the key features in hydrogel formation is that the biomaterials used should be able to self-assemble from a liquid monomeric phase to the solid polymeric mesh network under the influence of various factors such as pH, temperature, etc. Synthetic hydrogel preparations provide options for controlling structures and functions, with a wide range of mechanical properties. The biocompatibility of hydrogels is attributed to their similarity to the macromolecular-based components of the body [144]. Hydrogels are formed through covalent or non-covalent cross-linking of polymers [145]. A balance between adhesion of cells to the scaffold and the degradation rates of hydrogel tissue scaffolds is a key factor in promoting new tissue formation [146,147]. Therefore, an ideal hydrogel scaffold should have excellent degradation behavior, as also well-defined, reproducible, and tunable characteristics, so that it can be used in several applications, such as wound healing, cell differentiation, angiogenesis, etc. [148,149].

In skin tissue engineering, use of hydrogels such as hyaluronan–fibronectin hydrogels [2] and chitosan–gelatin hydrogels in combination with PLGA nanofibrous scaffolds [150,151] have been reported for wound healing and regeneration applications. Also, dextran-based hydrogels have provided complete skin regeneration during wound healing with efficient angiogenesis [152]. Use of hydrogel-based scaffolds for dermal and epidermal tissue regeneration looks promising, as reported in recent years [83,153,154,155,156]. A photo-crosslinkable gelatin hydrogel seeded with keratinocytes has been reported for its effective use as an epidermal substitute, wound dressings, and as a model for in vitro studies [156]. A rapidly transiting (viscous liquid into a solid scaffold) biohybrid hydrogel-collagen-glycosaminoglycan (GAG) has been reported to facilitate wound healing in rabbits [153]. In a study, poly(ethylene glycol)-g-chitosan (C-PEG) hydrogel permeated into a 3D porous chitosan–alginate (CA) scaffold was developed for creating a favorable bi-layered micro-environment to support fibroblasts on top and keratinocytes at the bottom [155]. Additionally, bilayer hydrogel scaffolds (either in combination with a fibrous mat or porous scaffold) have also been reported for effective drug delivery to enhance wound healing and to improve skin regeneration via seeding of stem cells derived from debrided human burn skin [157,158]. More recently, self-assembling peptide-based hydrogel scaffolds have been reported to accelerate burn wound healing and skin cells proliferation [159,160] with brighter prospects for dermal tissue regeneration. 

### 3.5. Microsphere Scaffolds

Scaffolding approaches using microspheres have garnered significant attention in recent years [161,162]. In advanced tissue engineering, microsphere scaffolds are primarily used for the effective delivery of drugs such as antibiotics or for gene therapy [163]. Incorporation of nanotechnology-based microspheres in scaffold design is showing promise for the development of sustained drug delivery, as drug encapsulated microsphere scaffolds are best suited for the release of drugs at a relatively slow rate for a prolonged period of time [164,165]. In microsphere scaffold design, which is generally a polymer mix, different drug delivery profiles can be achieved depending upon the molecular weight of polymers. For example, lower molecular weight results in more rapid release [166].

Microsphere scaffolding approaches offer several benefits, such as easy fabrication, controlled morphology and physicochemical properties, resulting in versatile use of the pharmacokinetics of the encapsulated molecules [167]. Microsphere scaffolds can be produced by heat sintering [168,169], treatment with solvent vapor [170,171], sintering with a solvent/non-solvent method [172,173] or only non-solvent sintering [162]. Microsphere scaffolds made up of several natural polymers have shown promise for development in bone and cartilage tissue engineering [174,175,176]. 

In the recent past, the use of various nanoparticle based microspheres, specifically PLGA, and natural polymers such as collagen or gelatin microspheres, has been reported for developing dermal or skin regeneration scaffolds for the effective delivery of drugs such as antibiotics or growth factors [177,178,179,180,181,182,183]. The size of microspheres in the scaffolds can be adjusted for the controlled release of proteins or drugs [177]. In a recent report, PLGA microsphere-based scaffolds were designed with a growth factor as well as gentamicin, and therefore effectively facilitated adhesion and proliferation of fibroblast cells with an antibacterial effect against Staphylococcus bacteria [183]. Also, mesenchymal stem cells-seeded microsphere scaffolds were successfully used as a skin construct and showed positive impact on cutaneous wound healing and sweat gland repair [184]. In addition, gelatin-based microsphere scaffolds have been reported for their use as microcarriers for stem cells, for skin regeneration [185]. All these reports highlight the utility of microsphere scaffolds for effective skin tissue regeneration. At the same time, though microsphere-based scaffolds have been reported for their use as a tissue regeneration remedies, biocompatibility of a few of these alginate microsphere-based scaffolds is still questionable as they impede wound re-epithelialization and cause inflammation [186].

### 3.6. Polymer–Bioceramic Composite Scaffold

The use of polymer–bioceramic composite materials for tissue engineering may be valuable, as it is advantageous in terms of controlling the properties of the material in order to provide favorable physiological responses of the host tissue [187]. There are mainly three categories of ceramics used in scaffold fabrication: (1) non-absorbable, which are inert in nature, e.g., alumina, zirconia, silicone nitrides, and carbons; (2) semi-inert surface reactive, e.g., glass ceramics and dense hydroxyapatite (HAP) (bio-reactive); and (3) non-inert biodegradable ceramics, such as aluminum calcium phosphate, coralline, plaster of Paris, HAP, and tricalcium phosphate (TCP) [188,189,190]. Use of ceramics has several merits, such as compatibility, resistance to corrosion and high compression. However, fragility, difficult fabrication, lack of reliability and resilience, and high density are some of the major disadvantages of ceramics. The term bioceramics is actually used to indicate the functions of ceramics in the field of regeneration of body parts, particularly bone. Polymers alone are flexible but have less mechanical strength and stiffness, whereas ceramics are stiff. Therefore, composite materials made up of polymers and bioceramics have improved mechanical properties, higher stiffness and strength. Additionally, polymer–bioceramic fabrication can significantly reduce the degradation (mainly of polymer) behavior of the scaffolds [191,192]. However, some of the challenges in designing polymer–bioceramic composite scaffolds include appropriate maintenance of strength and the stability, biocompatibility, bioactivity, and bio-resorption. All the same, highly porous polymer–ceramic composite scaffolding using PLGA/HAP provides excellent mechanical properties and biocompatibility [56].

In a recent review, use of bioceramic (usually bioactive glass) polymer-based scaffolds has been discussed for their application in soft tissue engineering [193]. Due to its angiogenic potential, bioactive glasses have created innovative outlooks in skin tissue engineering [194]. PLGA-mesh incorporated bioactive glasses (45S5 Bioglass^®^) have shown promising developments in the induction of neovascularization in scaffolds, both in vitro as well as in vivo [194]. Also, poly(3-hydroxyoctanoate)-fabricated nano-sized 45S5 Bioglass^®^ scaffolds have been reported for their application in wound dressing [195]. Such bioactive glass nanoparticles-based scaffolds are biocompatible in terms of providing a suitable environment for tissue growth (such as wettability and rough surfaces) and are useful in accelerating blood clot time [195]. Mesoporous bioactive glasses (MBG) fibers electro-spun with poly(ethylene oxide) have been reported to serve the dual purpose of supporting regenerated tissues and releasing anti-inflammatory drugs in skin tissue engineering [96]. Additionally, composite films made up of chitosan mixed with MBGs have a potential application for skin repair [196].

## 4. Biomaterials and Nanobiomaterials Used for Several Scaffolding Materials in Skin Tissue Engineering

Several natural, synthetic or composite biomaterials have been utilized for effective designing of scaffolding materials in skin tissue engineering. These biomaterials could be either microscale or nanoscale. In this review, we aim to discuss commonly used biomaterials in skin regeneration efforts, and we will also review the nanoparticle-based biomaterials that are being commonly used for developing scaffold-based dermal or epidermal substitutes. It is a well-known fact that nanotechnology has made a significant contribution to the development of suitable scaffolding material for tissue engineering. As of today, several biomaterials have been reported for their use in constructing tissue scaffolds. As described in the earlier part of this review, these biomaterials could either be natural or synthetic or a combination of both (composite scaffolds). Due to their resemblance to the natural ECM, biocompatibility, and biodegradability, natural polymers are widely used in wound and burn dressing. Natural polymers used in skin regeneration could be of protein or carbohydrate origin. Such polymers stimulate the healing by repair of the damaged tissue and promote effective skin regeneration [197]. On the other hand, synthetic polymers are fabricated (mainly using electrospinning) with controlled degradation characteristics and architecture [198,199,200].

### 4.1. Natural Biomaterials of Protein Nature

In the field of skin tissue engineering, the commonly used natural biomaterials which are mainly produced as proteins are collagen, gelatin, silk and fibrinogen. Being an integral part of the ECM, collagen offers tensile strength for tissue growth. It is produced by fibroblasts to stimulate faster wound healing and is the most abundant biomaterial of natural protein produced not only in the skin tissue but overall in the human body. Collagen protein is a helical polypeptide with the repeating sequences of glycine, proline and hydroxyproline [8,201,202]. Nearly 28 different types of collagen have been identified [8,201] out of which collagen type I is predominantly a part of the ECM of skin, tendon and bone. Minor amounts of collagen type III are found in skin. The adhesion domains of fibrillar collagen (50–500 nm) in ECM promotes cell adhesion and proliferation [8,203,204]. Several animal origin (bovine, porcine and avian) collagen dressings have been formulated and are effective in the repair of wounds to full-thickness with a contraindication for third-degree skin burns and for allergic conditions [205]. Collagen-based acellular (Integra^®^ and Brisbane^®^) and cellular (Apligraf^®^ and Transcyte^®^) skin substitutes are effective in accelerating wound healing by supporting an appropriate environment for fibroblast and keratinocyte proliferation [8,203]. Numerous studies have reported different types of collagen dressing formulations for wound and burn repair including collagen sponges for deep skin wounds [206,207], including Glycagen^®^ (collagen–glycosaminoglycan complex) [1], collagen absorbable membrane [208], collagen composite films [209], composite of type III collagen with polysaccharides [210,211], drug loaded collagen hydrogels [212], microfiber collagen scaffolds [213], and electrospun collagen nanofibrous scaffolds [214]. Collagen nanofibrous scaffolds have been developed as skin substitutes by various electrospinning approaches, such as electrospinning of collagen type I and type III [84], controlling the alignment of collagen nanofibers [215], solvent electrospinning of collagen [8], coating of scaffolds with collagen instead of solvent electrospinning [216] and cross-linking of collagen [8]. Another natural biomaterial is gelatin, which is a partially hydrolyzed version of collagen wherein the triple-helical structure of collagen is changed into single-stranded molecules [217]. Gelatin is advantageous over collagen as it is less immunogenic and allows enhanced cell adhesion due to the presence of arginine-glycineaspartic acid (RGD) sequences [218,219]. Scaffolds made of gelatin nanofibers using the electrospinning method have shown potential applications in wound healing processes [8]. Various formulations of gelatin wounds and burns dressing, such as gelatin-alginate sponges [220], gelatin containing EGF [221] and gelatin films, have shown potential applications in the treatment of wound and burn skin tissue.

Silk is a biomaterial mainly produced by silkworms and is a classical fibrous protein in nature. Out of the two kinds of silk proteins, i.e., fibroin (hydrophobic) and sericin (hydrophilic), biodegradable fibroin is highly biocompatible with minimum inflammatory reaction and provides great permeability for nutrients and therefore has been widely used to engineer scaffolds for tissue engineering applications [222,223,224]. Silk fibroin (SF) based scaffolds prepared by electrospinning have garnered much attention as wound dressing materials as they promoted better spreading of collagen and cell adhesion on their surface compared to a simple SF film or a woven matrix of SF microfibers [225]. Due to high biocompatibility, flexibility and minimum inflammatory reaction, SF is very useful in wound dressings and skin grafts [1]. SF based scaffolds, nanofibers, sponges or membranes and cytocompatible porous SF films have shown promising results in wound healing and skin tissue regeneration [1,226]. A glycoprotein, fibrinogen, is an important blood coagulation factor produced by the liver and has a key role in wound healing [227]. Fibrinogen is converted into fibrin, a fibrous, non-globular protein, and fibrin matrix-based scaffolds are widely used in several skin regeneration products [227,228]. Natural scaffolds made up of fibrin and anti-inflammatory bandages composed of thrombin and fibrinogen are used for wound dressing and skin regeneration applications [229,230].

Numerous biomaterials of animal origin, such as keratin, bovine serum albumin, egg shell membrane proteins, etc., have been used effectively in skin regeneration products. Keratin and its derivatives have been used in various dressing materials to either release antibiotics or growth factors and thus are useful in wound healing applications [231]. Keratin is an intermediate filamentous protein found abundantly in tissues, such as horns, claws and hooves [232]. Nanofibers made up of bovine serum albumin (BSA) have potential application in wound closure suturing materials as these nanofibers are highly biocompatible and biodegradable [233]. Avian egg shell membrane which is a meshwork of fibrous proteins, is used in the treatment of wounds and burn injuries as they provide an ECM environment for human dermal fibroblast cells [234]. Gastric pentadecapeptide has been used topically (cream) or systemically [intra-peritoneal (i.p.) injections], with improved wound healing compared to untreated controls. A pentadecapeptide BPC 157 obtained from human gastric juice has shown improved healing of burn-wound and gastrointestinal lesions [235]. Similarly, growth factors are the proteinous biomaterials used mainly as composite biomaterials in combination with synthetic polymers and play a major role in stimulating the cell proliferation to promote effective wound healing [1]. Few of the enzymes, such as collagenase, papain and lysostaphin (Lst), have lytic effect and are useful for biochemical debridement of the wounds [1,236].

In addition to all these biomaterials of protein nature, some vegetable proteins in combination with polysaccharides such as soya protein cross-linked with glutaraldehyde and sago starch are effective in wounds and burns dressing [1,237].

### 4.2. Polysaccharide Natural Biomaterials

Polysaccharides-based biomaterials, which are mainly used in the form of hydrogels for the effective management of skin wounds and burns, can be categorized as neutral (e.g., glucans, dextrans, cellulose), acidic (alginic acid and hyaluronic acid), basic (chitosan) or sulfated polysaccharides (heparin, chondroitin) [1]. The most popular and naturally produced biomaterials of polysaccharide origin are chitosan, hyaluronic acid and alginate. These polysaccharide biomaterials can also be sub-dived as homoglycan polysaccharides such as glucans, cellulose, dextran and chitosan; and heteroglycan polysacchrides such as alginates, agarose, carrageenans, pectins, gums, glycosaminoglycans, all of which exhibit peculiar physicochemical properties and stronger biocompatibility and biodegradability and thus have important applications in biomedical fields [1]. A non-toxic and super-absorptive hydrogel made up of pullulan (glucan polysaccharide), a biosynthetic derivative of starch, has been effectively used for antibacterial or antimycotic drugs released at wound site [238]. Different types of d-glucans derived from yeast, grain, and fungi have been utilized to prepare the gel structures which support effective wound healing with lowered skin irritation [239]. A dextran biomaterial, Carboxymethyl Benzylamide Sulfonate Dextran (CMDBS), has structural similarity with the glycosaminoglycan heparin, and is well-known for not only stimulating wound healing but also for being effective in controlling *Staphylococcus aureus* biofilm formation [240]. Bioengineered cellulose is popularly used as a healing scaffold/matrix for dressing of partial or full thickness chronic wounds as it stimulates the granulation and epithelialization. Cellulose of bacterial origin (*Acetobacter xylinum*) is a unique nanostructured biomaterial and has a great potential for wound dressings and tissue engineered skin [241,242]. Being a non-toxic and biodegradable derivative, cellulose is widely used to design the wound healing scaffold for severely damaged skin due to its similarity with ECM [241,243]. Porous nanofibrous cellulose membranes have several applications in tissue repairing and remodeling, or skin transplantation [244]. 

Another homoglycan polysacchride, chitosan, which is a derivative of an important component of arthropod exoskeleton and crustacean shells called chitin, is the most popular polysaccharide biomaterial. Chitosan is composed of β (1–4) linked d-glucosamine residues with randomly distributed *N*-acetyl-d-glucosamine groups [245]. Chitosan has a wide range of applications in burn and wound treatments due to its hemostatic, antimicrobial and antifungal properties [245,246]. Chitosan accelerates natural blood clotting by activating platelets, thus helping faster wound healing. It also stimulates proliferation of fibroblasts, cytokine production through macrophage activation and angiogenesis. Besides this, chitosan also promotes synthesis and deposition of two important biomaterials of ECM, including collagen (through the gradual release of depolymerizied *N*-acetyl-d-glucosamine) and hyaluronic acid at the wound site. Thus, chitosan not only provides a wound healing effect by itself but also, in turn, helps in faster and scar-free wound healing by producing other important biomaterials [8]. In spite of a few challenges in preparing chitosan-based scaffolds due to its ionic characteristics, chitosan nanofibrous scaffolds were reported to offer better adhesion, proliferation and differentiation of keratinocytes, fibroblasts and endothelial cells, as well as enhanced vascularization and formation of granulation tissue compared to two dimensional (2D) films and 3D sponges of chitosan [8,247,248]. There are several different chitin/chitosan formulations reported for wound healing properties, such as water-soluble chitin (WSC) ointment [1], partially deacetylated chitin hydrochloride [249], chitin and silver nanoparticles/silver sulfadiazine/nano zinc oxide composites [250,251], phosphorylated chitin and chitosan, co-cultured keratinocyte and fibroblast cells on chitin/chitosan hydrogel membranes and scaffolds, antimicrobial films, sponges and hydrogels of chitosan [1], chitosan mesh membrane [252], chitosan films with antioxidant or thyme oil [1,253], and chitosan–Aloevera membranes [254]. 

Among heteroglycan polysaccharide based biomaterials, agar, natural agarose fibers and carrageenan hydrogels are promising bio-materials for wound dressing applications [1,255]. Hydrocolloids, such as pectins and gums, have also shown potential applications as occlusive and semi-occlusive moist dressings materials for wounds and burns [1]. Another biomaterial of heteroglycan polysaccharide origin is water soluble alginate, which is composed of repeating units of α-l-guluronate and β-d-mannuronate and is derived from marine brown [8]. Water solubility of alginate is beneficial for the absorption of wound exudate and permits the maintenance of the moist wound environment [256]. Electrospun scaffolds based on alginate have been reported for their use in skin tissue regeneration [8,256]. Alginate-based porous and non-adhesive wound dressings along with secondary dressing materials are commonly used for wounds and burns induced skin injuries due to their hemostatic properties [1]. Due to the high water absorbing capacity of alginate, a suitable moist environment is maintained at the site of the injury [1,257]. 

Another important natural biomaterial used in current commercial wound healing products (such as Hyaff^®^, Laserskin^®^, and Hyalograft^®^) is hyaluronic acid (HA), a glycosaminoglycan poylsaccharide [258]. Glycosaminoglycans such as HA, heparin and chondroitin sulfate are essential for skin regeneration as they are the most important components of the ECM [259]. HA is one of the main components of the ECM of connective tissues [260]. It is composed of repeating glucuronic acid and *N*-acetylglucosamine chains and has several important functions, such as fast wound healing without scar formation, enhancing mitotic division of epithelial cells, and regulating macrophages to adjust the phagocytosis mechanisms [261]. HA hydrogel scaffolds are well-known for directing tissue regeneration by supporting angiogenesis and neuritis outgrowth/repair [262]. On the other hand, high viscosity and surface tension that leads to enhanced water capacity, limits the use of HA in scaffold designing due to poor electrospinning [8]. This, however, can be solved by creating HA nanofibers via the air blowing technique, wherein evaporation of solvent occurs due to hot air leaving behind consistent nanofibers [88]. These HA nanofibrous materials have shown faster wound healing in pigs compared to HA adhesive bandage or vaseline-based gauge bandage [261]. Similarly, heparin-coated aligned nanofiber scaffolds are helpful for increased endothelial cell infiltration in full-thickness dermal tissue remodeling [263], and heparin sulfate combined with a polymer, OTR4120, decreases inflammation and stimulates angiogenesis and collagen maturation in the skin regeneration process [264].

### 4.3. Synthetic Biomaterials

Considerable research has been performed to develop self-assembling and biomimetic synthetic biomaterials [8]. Most of the synthetic biomaterials are either biodegradable or non-biodegradable. Among the biodegradable biomaterials, aliphatic polyesters are the most common and are well-known materials due to their high mechanical strength, flexible properties and easy processability, and more importantly their non-toxic degradation [8,265]. Polylactic acid (PLA), polyglycolic acid (PGA), polycaprolactone (PCL) and their copolymers are some of the FDA approved-aliphatic polyesters which have been extensively used in wound dressing and skin tissue regeneration products. Most of the nanomaterial-based synthetic polymers used for development of skin wounds dressings are prepared by electrospinning [8,266]. Electrospun polyvinylpyrrolidone blended nanofibrous membranes have been shown to be effective in drug delivery and accelerated wound healing applications [267]. Similarly, films made up of fatty acid based polyurethane, and biodegradable poly-3-hydroxybutyrate-poly-ε-caprolactone have shown promising results in wound dressing [150,268]. Also, poly-ε-caprolactone homopolymers and poly-l-lactide-ε-caprolactone matrices have potential applications in tissue repair [1]. In a sheep model for dermal wound healing, dressing material, made up of silicone-coated non-woven polyester, enhanced re-epithelialization at the wound site [269]. In addition, all the synthetic materials in combination with the natural polymers have been extensively used in skin tissue regeneration and are discussed in the section below as composite biomaterials.

### 4.4. Composite Biomaterials

Composite biomaterials have been widely used in skin tissue engineering for wound healing and repair applications. The composite mixture could either be made up of different natural or synthetic polymers or a combination of both. A composite of the vegetable proteins in combination with polysaccharide, such as cross-linked soya protein with sago starch have been reported to be effective in wounds and burns dressing [1]. Another composite mixture of soy protein, with sodium caseinate-based membranes is highly biocompatible and biodegradable and has potential application in drug delivery and wound dressing [237]. Several composite mixtures suitable for skin or dermal tissue regeneration applications include chitosan nanoparticles containing fibrin gels [270], thrombin receptor agonist peptide (TRAP) encapsulated in poly(*N*-vinyl caprolactam)-calcium alginate (PVCL) hydrogel films [271], biopolymeric matrices delivering angiogenic growth factors [272], or epidermal growth factor (EGF) delivering micro- and nanoparticulates [273]. Composites made up of cellulose-chitosan and cellulose-poly(methylmethacrylate) fibers have been used as anti-infective bandages with specific bactericidal activity against *S. aureus*, which is attributed to the cell lytic enzyme, lysostaphin (Lst) [236]. Cellulose-based composite wound dressing materials contain different active molecules, such as enzymes, antioxidants, hormones, vitamins and antimicrobial drugs [1]. Similarly, cellulose of microbial origin along with chitosan exhibits better biocompatibility than cellulose alone, in terms of cell adhesion and therefore the composites are used effectively for wound dressing and tissue engineering [1]. In the recent past, microbial cellulose has been used to prepare different composite sheets with montmorillonite (MMT) [1]. The composite films made up of microbial cellulose and MMT have stronger antibacterial properties and a therapeutic importance in wound healing and tissue regeneration. There are plenty of chitosan-based composite biomaterials which have potential applications in wound healing and tissue regeneration, such as films made up of chitosan–cellulose–silver nanoparticle mixtures, chitosan–gelatin spongy mixtures, chitosan gelatin–antibiotic mixtures [1], chitosan–alginate polyelectrolyte membranes [274], chitosan gels containing EGF [275,276], tencel–chitosan–pectin composite [1], chitosan–fibrin nanocomposites like nanofibrous chitosan–silk fibroin composite, beads of chitosan–fibrin, and chitosan–fibrin–sodium alginate [1].

Different alginate-based materials, such as zinc alginate and silver alginate have tissue healing applications due to their antimicrobial properties [1]. Some of the alginate-based composite biomaterials include alginate films loaded with asiaticoside and alginate–chitosan membranes [1].

Composite hydrogels of poly(*N*-vinyl-2-pyrrolidone) (PVP), kappa-carrageenan (KC), potassium chloride, and polyethylene glycol and PVP-KC have been reported for their applications in tissue engineering [255]. Such hydrogels were prepared by exposure to higher doses of gamma-radiation and then evaluated for their wound dressing applications [255]. Other hydrogel membranes made up of pectin and gelatin have applications in wound dressing [1]. Several composite mixtures of natural polymers along with antibacterial components, such as a natural polymer derived from a fruit gum, fragrant manjack, snotty gobbles (*Boraginaceae*) with different percentages of glycerin [1], extracellular polysaccharide of *Trametes versicolor*, polymer of fungal origin, have shown great potential for the treatment of wounds with desired antibacterial effects. 

Composite polyurethane foams impregnated with HA and silver sulfadiazine have been shown to reduce wound size significantly in an experimental rat model [277]. Another HA-based composite hydrogel for tissue repairing application is developed by either crosslinking of HA with glycidyl methacrylate groups and DNA [278], or functionalization of HA with thiol cross-linking sites [279]. Similarly, composite mixtures such as glycolipids, and proteoglycans have also been reported for their effectiveness.

Glycolipids, such as galactose liposomes, have some potential application in skin wound repairs in the experimental knockout mice model [280]. Proteoglycans are composed of proteins and glycosaminoglycan. Among the several reported proteoglycans, neoproteoglycans can be categorized as conjugates of protein and glycosaminoglycan, nanoparticles conjugated-glycosaminoglycan and polymer conjugated-glycosaminoglycan [281]. Several composite biomaterials synthesized using either natural or synthetic biomaterials have antibacterial components incorporated, such as polyurethane–dextran mats with ciprofloxacin [282], cellulose acetate and shikonin containing poly-l-lactide and poly(lactide-*co*-glycolide) [283], chitosan–poly(*N*,*N*-diethyl-acrylamide) films, scaffolds of poly(ethylene glycol)/chitosan loaded with ciprofloxacin, silver nanoparticles along with chitin-based films [1,284]. Several PEG based composite mixtures have also been reported for wound healing purposes, such as heparin functionalized PEG [285], a combination of proteins with PEG [286] and gelatin-PEG [287].

Sponges made up by crosslinking gelatin with various materials have also been used effectively [288]. Polyvinyl alcohol-based composite mixes with gelatin or carboxymethyl cellulose [283,289,290], polyacrylic acid [291], and polyethylene glycol have promising wound healing effects. Polylactic acid-based composites with curcumin and collagen are also emerging as useful biodegradable materials in wound dressing materials [1]. Composite polyurethane membranes and films have shown excellent absorptive and antibacterial properties for wound dressing materials [292]. Polyvinylpyrrolidone-based composite materials have also shown promising dermatological applications [1].

## 5. Conclusions

As of today, several types of scaffolds have been reported in skin tissue regeneration. Despite some of their drawbacks, these scaffolds have provided remarkable success in skin tissue repair and wound healing. Scaffolding provides a suitable 3D environment for the cells to grow, proliferate and differentiate. Additionally, due to varying degrees of porosities, scaffolds provide an excellent vehicle for the cells for the regular supply of nutrients and oxygen. Although mechanical strength and biocompatibility are the major concerns for the fabrication of scaffolds, composite or bioceramic scaffold development has good future prospects. Various natural and synthetic biomaterials are used to create the scaffolding materials either in combination or per se. The combination of such materials resolves around issues such as biocompatibility, biodegradability and mechanical strength. Natural biomaterials, such as collagen, cellulose, chitosan, etc., are either protein or polysaccharide in nature. Due to their close resemblance with the natural ECM, they are highly biocompatible and easy to degrade and thus are best suitable for skin cell growth. On the other hand, synthetic biomaterials including various types of nanomaterials, such as polyvinylpyrrolidone (PVP), polycaprolactone (PCL), poly-ethylene-glycol (PEG), poly lactic acid (PLA), etc., are good in enhancing the strength of the scaffold material. Therefore, more efforts towards developing composite scaffolds for skin tissue growth are required. Appropriate knowledge of the above facts will enable the production of suitable scaffolds for skin tissue regeneration applications. At the same time, some of the leading challenges in the field of skin tissue engineering, such as cells to scaffold interaction, faster cellular proliferation and differentiation, as well as vascularization of the engineered tissues remain to be overcome. Similarly, with the rapid advancement of the organ-on-chip field, efforts directed towards the development of “skin-on-chip” technology have shown remarkable promise for generating engineered skin for wound healing or drug testing [293]. Recent success has been achieved through the development of perfused chip-based bioreactors which offer variable mechanical stress and improve the culturing conditions for skin organ cultures. At the same time, microfluidics technologies intended for developing perfused skin-equivalent cultures show promise in the clinical application of various drug molecules particularly associated with skin tissue wound healing. 

## Figures and Tables

**Table 1:** Advantages and disadvantages of different scaffolds used in skin tissue engineering.

Scaffold Types	Advantages	Disadvantages	Future Prospects
Porous scaffolds	High porosity provides a suitable environment for extracellular matrix (ECM) secretion and nutrient supplies to the cells. Pore sizes specific to the cell types prevent clustering of the cells, thus avoiding necrotic center formation.	Porous nature limits the homogenous distribution of the cells. Different pore sizes are required for the specific cell types and are therefore time consuming.	Improvement in the connectivity of pores and thereby the structure of the scaffolds is required.
Fibrous scaffolds	Highly microporous structure is best suitable for cell adhesion, proliferation and differentiation. Low inflammatory response upon implantation.	Surface functionalization is required to create the nanofibers of these scaffolds.	Drugs and biological molecules such as proteins, genes, growth factors, etc., can be incorporated in fibrous scaffolds for release applications.
Hydrogel scaffolds	Highly biocompatible and controlled biodegradation rate.	Limited mechanical strength due to soft structures.	Degradation behavior of the hydrogels and tenability should be well-defined. Hydrogels incorporating growth factors to facilitate cell differentiation.
Microsphere scaffolds	Easily fabricated with controlled physical characteristics suitable for slow or fast drug delivery. Provides enhanced cell attachment and migration properties.	Microsphere sintering methods are sometimes not compatible to the cells and reduces the cell viability.	These scaffolds can be used as a target specific delivery vehicle for the drugs such as antibiotics, anti-cancer, etc.
Composite scaffolds	Highly biodegradable and offer mechanical strength. Greater absorbability.	Acidic byproducts are generated upon degradation. Poor cell affinity. Require tedious efforts to develop composite scaffolds.	Nano-bioceramic and polymer composites with faster degradation are currently being developed.
Acellular scaffolds	Native ECM is retained and thus normal anatomical features are maintained. Less inflammatory and immune response with higher mechanical strength.	Incomplete decellularization is required to avoid immune responses.	Such scaffolds hold promise towards developing artificial organs.

## Abbreviations

CA	chitosan–alginate
HA	hyaluronic acid
ECM	extracellular matrix
PBT	Polybutylene terephthalate
PCL	Polycaprolactone
PDLLA	poly(d,l-lactic acid or d,l-lactide)
PEE	polyester elastomer
PEO	polyethylene oxide
PEG	Polyethylene glycol
PGA	polyglycolide
PLA	poly(lactic acid)
PLGA	poly(lactic-*co*-glycolic acid)
PTFE	polytetrafluoroethylene
PVP	poly(*N*-vinyl-2-pyrrolidone)
SF	Silk fibroin
